# Thermophysical Properties of Hybrid Nanofluids and the Proposed Models: An Updated Comprehensive Study

**DOI:** 10.3390/nano11113084

**Published:** 2021-11-16

**Authors:** Mohammad M. Rashidi, Mohammad Alhuyi Nazari, Ibrahim Mahariq, Mamdouh El Haj Assad, Mohamed E. Ali, Redhwan Almuzaiqer, Abdullah Nuhait, Nimer Murshid

**Affiliations:** 1 Institute of Fundamental and Frontier Sciences, University of Electronic Science and Technology of China, Chengdu 610054, China; m_Nazarii@uestc.edu.cn; 2 College of Engineering and Technology, American University of the Middle East, Kuwait; Ibrahim.Maharik@aum.edu.kw (I.M.); nimer.murshid@aum.edu.kw (N.M.); 3 Sustainable and Renewable Energy Engineering Department, University of Sharjah, Sharjah P.O. Box 27272, United Arab Emirates; massad@sharjah.ac.ae; 4 Mechanical Engineering Department, College of Engineering, King Saud University, P.O. Box 800, Riyadh 11421, Saudi Arabia; ralmuzaiqer@ksu.edu.sa (R.A.); anuhait@ksu.edu.sa (A.N.)

**Keywords:** hybrid nanostructures, thermophysical features, dynamic viscosity, thermal conductivity

## Abstract

Thermal performance of energy conversion systems is one of the most important goals to improve the system’s efficiency. Such thermal performance is strongly dependent on the thermophysical features of the applied fluids used in energy conversion systems. Thermal conductivity, specific heat in addition to dynamic viscosity are the properties that dramatically affect heat transfer characteristics. These features of hybrid nanofluids, as promising heat transfer fluids, are influenced by different constituents, including volume fraction, size of solid parts and temperature. In this article, the mentioned features of the nanofluids with hybrid nanostructures and the proposed models for these properties are reviewed. It is concluded that the increase in the volume fraction of solids causes improvement in thermal conductivity and dynamic viscosity, while the trend of variations in the specific heat depends on the base fluid. In addition, the increase in temperature increases the thermal conductivity while it decreases the dynamic viscosity. Moreover, as stated by the reviewed works, different approaches have applicability for modeling these properties with high accuracy, while intelligent algorithms, including artificial neural networks, are able to reach a higher precision compared with the correlations. In addition to the used method, some other factors, such as the model architecture, influence the reliability and exactness of the proposed models.

## 1. Introduction 

The heat transfer capacity of single-phase fluids, including water, oil and ethylene glycol, is relatively poor due to their low thermal conductivity. Since the intensification of heat transfer in industries and power plants is very important from both technical and economic points of view, a new generation of fluids with solid nanostructures has been recently applied. These fluids, known as nanofluids, are made of a base fluid and suspended solid particles, sheets or tubes in nano dimensions [1]. There are different approaches for the preparation of nanofluids. Physical methods, such as ball milling and ultrasonication, in addition to chemical processes, such as functionalization, are among the main approaches used for the preparation of nanofluids [2]. Metal oxide particles, such as CuO, Al_2_O_3_, TiO_2_ in addition to the carbonic materials, including Carbon Nano Tubes (CNTs), graphene and graphite, are among the most commonly used nanostructures in the nanofluids that are applied as heat transfer fluids [3,4]. By applying functionalization, it would be possible to modify some properties of CNTs, such as solubility, that makes them more dispersible in the base fluid [2]. The dispersion of solid materials with nanometer dimensions in the fluid can modify their heat transfer properties [5]. Improvement in these properties is dependent on different factors that are considered in various researches [6,7,8]. Regarding the improved heat transfer characteristics of nanofluids, they can be applied to enhance the performance of various systems, such as thermoelectric generator modules and heat exchangers [9,10,11]. In addition to the single type nanostructures, two or more dissimilar nanomaterials could be added to the base fluid, which leads to the preparation of hybrid nanofluids [12]. The idea of employing hybrid nanofluids is to achieve more enhancement in thermal characteristics and pressure drop by a trade-off between the disadvantages and advantages of an individual nanostructure, which is attributed to a more proper thermal network, appropriate aspect ratio and synergistic impact of nanostructures [13]. In other words, hybrid materials simultaneously combine the chemical and physical properties of two or more materials that provide these specifications in a homogenous phase [13]. These properties may not be obtained in the case of using an individual nanomaterial. Several studies have shown that hybrid nanofluids have improved thermophysical properties in comparison to the nanofluids containing a single material; however, some of the studies have reported that the thermal conductivity of hybrid nanofluids is lower than conventional ones, which is mainly attributed to the non-compatibility of the materials with each other, inappropriate size of the nanostructures, stability of the nanofluid and temperature of the fluid [14]. In this regard, to prepare a hybrid nanofluid with desirable properties, it is crucial to consider different factors. Similar to mono nanofluids, different approaches are applicable for the synthesis and preparation of hybrid nanofluids, which are dependent on the properties of the composites and required stability.

Hybrid nanofluids, due to their modified thermal characteristics, have been tested in numerous thermal engineering applications [15,16,17,18]. For instance, Hussein et al. [19] applied a hybrid nanofluid composed of covalent functionalized multi-walled carbon nanotubes (MWCNTs) and covalently functionalized graphene nanoplatelets in a flat plate solar collector. They observed that utilizing the hybrid nanofluid in a collector caused around 20% higher efficiency of the system in comparison with the case of using distilled water. Fattahi [20] investigated the performance of a hybrid nanofluid in a solar collector and concluded that up to 23% improvement in the heat transfer was possible by employing the nanofluid. Asokan et al. [21] investigated the effect of using nanofluids containing CuO, Al_2_O_3_ and their hybrid particles in a compact heat exchanger. They found that applying the hybrid nanofluids caused better enhancement in the performance compared with the mono nanofluids. Pandya et al. [22] charged an axial grooved heat pipe with a hybrid nanofluid composed of CeO_2_-MWCNT and observed that using this working fluid led to 30% lower thermal resistance compared with the case of using water as the working fluid. Aside from the abovementioned applications, using hybrid nanofluids in other types of thermal mediums, such as pulsating heat pipes, could result in the improvement of performance [23]. 

Among thermophysical properties of fluids, thermal conductivity, specific heat and dynamic viscosity play key roles in their heat transfer characteristics. Although there are some review articles on the thermophysical properties of nanofluids, there are few works that consider both the properties and the proposed models. In addition, the majority of the review papers have focused on one or two properties of nanofluids, mainly TC and DV, while specific heat is another property that influences the heat transfer characteristics of nanofluidic flow. In this regard, the present work focuses on all of the mentioned properties of hybrid nanofluids as promising heat transfer fluids. Compared with other studies on similar topics, the present work is updated and considers all of the recently published documents. In the following sections, studies on these thermophysical properties and the corresponding models are reviewed. Furthermore, some recommendations are proposed for the upcoming studies in the related research. 

## 2. Methodology

As mentioned in the introduction section, heat transfer characteristics of nanofluids are significantly affected by three thermophysical properties, including thermal conductivity, dynamic viscosity and specific heat. The aim of the present paper is to review the factors affecting these features and the proposed models used for predicting and estimating these properties of nanofluids. In this regard, different search engines, including SCOPUS, Google Scholar and EBSCO, and other sources, such as the websites of publishers, including MDPI, ELSEVIER, Wiley, Taylor & Francis, Springer Nature, ASME and OXFORD, were applied to search the keywords. The main keywords used for the search were “Hybrid Nanofluids”, “Thermal Conductivity”, “Dynamic Viscosity”, “Specific Heat”, “Machine Learning” and “Artificial Neural Network”. In the first step, to find the sources for the properties of the nanofluids, Boolean AND was used to join “Hybrid Nanofluid” with one of the following keywords, including “Dynamic Viscosity”, “Thermal Conductivity”, “Specific Heat”. Afterward, Boolean AND was used to join other keywords. Other keywords that were used for the studies on the forecasting and prediction of these nanofluids were “Modeling”, “Artificial Intelligence”, “Neural Network” and “Machine Learning”. In this case, Boolean OR was applied to find all of the works on the mentioned properties. All documents published between 1990 and 2021 were considered for evaluation. It should be mentioned that just English documents were used for the review. The first inclusion criterion for the consideration of the sources for review was the consideration of hybrid nanofluids for investigation. The second inclusion criterion was the consideration of one of the three mentioned thermophysical properties. The exclusion criteria consisted of all of the studies that did not meet the mentioned criteria. It should be mentioned that review papers were considered for the introduction section. Both experimental and numerical works were considered for the present work in order to provide a comprehensive study. Afterward, two of the authors checked the gathered documents independently to select the appropriate ones. They considered the abovementioned criteria to include the proper documents for the current review. 

In cases where there was disagreement between the referees, a third one was asked to finalize the decision. Subsequently, the authors categorized the documents on the basis of the properties. In this regard, the selected documents were divided into three main groups, including thermal conductivity, dynamic viscosity and specific heat. Afterward, the categorized documents for each property were divided into two groups, including the models and measurement. Subsequently, the main findings of the selected documents were summarized to be reflected in the current article. 

The most important questions to be answered in this review paper are as follows:

- Which factors affect the thermophysical properties of the hybrid nanofluids?
- How do the influential factors affect the properties of hybrid nanofluids?
- How can we model and forecast the thermophysical properties of hybrid nanofluids?
- Which factors influence the accuracy of the models used for the properties of the hybrid nanofluids?
- How can we improve the exactness of the proposed models?

## 3. Thermal Conductivity

**Figure 1:**
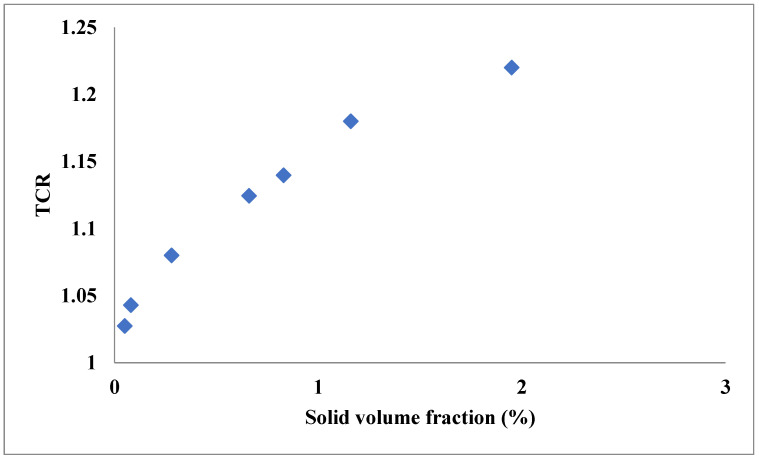
Effect of solid VF and temperature on TCR of SiO_2_-MWCNT (85:15%)/EG. Adapted from Ref. [35].

**Figure 2:**
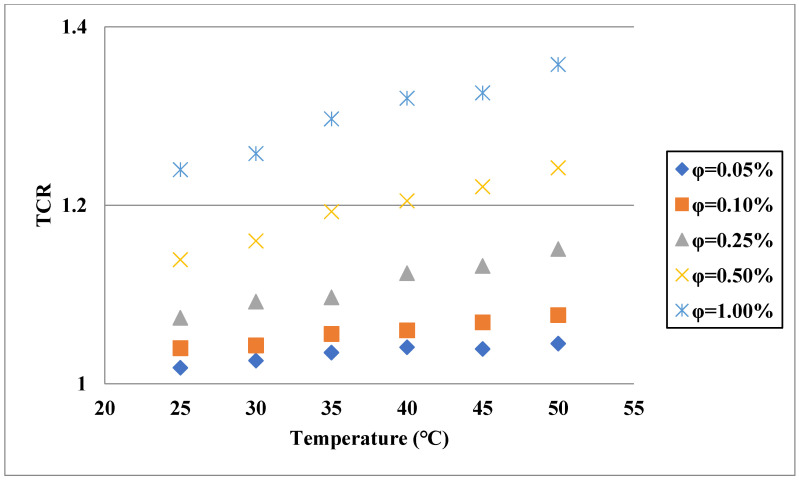
TCR and the enhancement in TC for different VFs. Adapted from Ref. [37].

Thermal conductivity (TC) of the fluids plays a crucial role in their heat transfer ability; in this regard, it is desirable to utilize fluids with higher thermal conductivity. The dispersion of hybrid solids in nanometer dimensions can notably improve the TC owing to the Brownian motion, intermolecular interaction of the nanostructures, higher TC of solids compared with the liquids and clustering of the nanostructures [24,25,26,27,28]. The enhancement rate of hybrid nanofluid TC depends on some factors, such as the temperature and volume fraction (VF) [29,30,31,32,33,34]. For instance, Esfe et al. [35] measured the TC of a hybrid nanofluid composed of SiO_2_-MWCNT and ethylene glycol (EG) at different temperatures and VFs of the solid phase. They found that the increase in the temperature and solid VF caused an increase in the thermal conductivity ratio (TCR). They found that in the considered ranges of VF (0.05–1.95%) and temperature (30–50 °C), the highest value of the increase in TCR was 22.2%, as shown in Figure 1. The increase in the TC with temperature is mainly attributed to the Brownian motions of the nanostructures, while increases in the TC with VF are mainly due to higher TC of the solid materials compared with the base fluids. In another work [36], the influences of temperature and VF on the TC of WO_3_-MWCNT/engine oil were investigated. The ranges of VF and temperature were 0.05–0.6% and 20–60 °C, respectively. It was found that despite the increase in the TC by increasing VF and temperature, the effect of VF was more notable. The highest enhancement in the TC of the investigated hybrid nanofluid was 19.85% compared with the base fluid that was obtained at the highest temperature and VF. Higher enhancement in the TC of hybrid nanofluids has been observed by using other nanostructures. Utilizing hybrid material containing carbonic-based materials, such as graphene, graphite and CNTs, can lead to significant enhancement in the TC. As an example, Kazemi et al. [37] measured the TC of graphene-SiO_2_/water in various conditions. As shown in Figure 2, it was observed that around 36% enhancement in TC was reachable at 50 °C and VF of 1%. Esfe et al. [38] measured TC of SiO_2_-DWCNT/EG in various temperature and VF ranges of 30–50 °C and 0.03–1.71%, respectively. The highest improvement in the TC of nanofluid compared with EG as the base fluid was 38%. In another study, Pourrajab et al. [39] found that the existence of 0.04% vol and 0.16% vol MWCNT in water could improve the TC by up to 47.3% compared with water as the base fluid. Trinh et al. [40] measured the TC of Gr-CNT/EG nanofluid at different temperatures and observed that it was enhanced by 50% at 50 °C in solid VF of 0.07%. 

**Figure 3:**
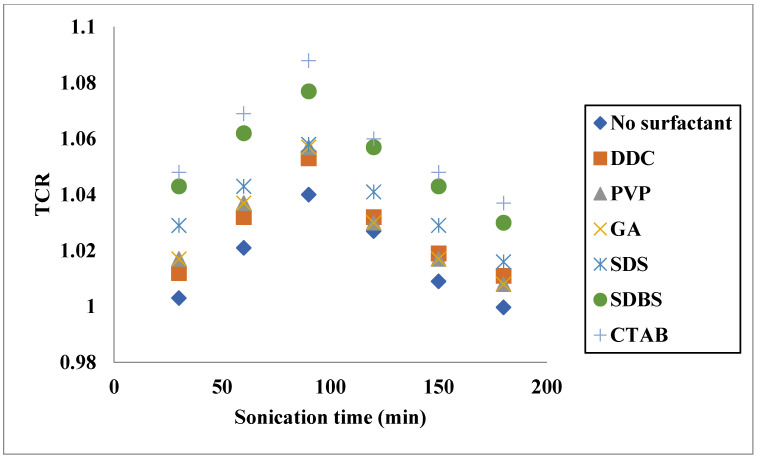
Effects of surfactant and sonication time on TCR of CeO_2_-MWCNT/water (SMR: surfactant mixing ratio = 3:2, φ = 0.75%, T = 30 °C). Adapted from Ref. [42].

Aside from the temperature and VF of solid parts, other factors are involved in the TC enhancement of hybrid nanofluids. As an example, Dalkılıç et al. [41] measured the TC of CNT-SiO_2_/water nanofluid under different conditions. In addition to the temperature and VF of nanostructures, the mass ratio of solid structures was varied to investigate its effect on TC. It was noticed that the highest enhancement of TC obtained for VF of 1%, and mass ratio of 0.8 CNT and 0.2 SiO_2_ and was equal to 26.29%, while the minimum enhancement was noticed for 0.1% VF and 0.2 CNT and 0.8 SiO_2_ that was equal to 0.78%. In another work, impacts of other variables, such as sonication duration and surfactant on the TC of a hybrid CeO_2_-MWCNT/water nanofluid, were investigated by Tiwari et al. [42]. They found that there was an optimal sonication time to reach the maximum TCR. Furthermore, they noticed that the surfactant could influence the TC, as shown in Figure 3. The mixture ratio, indicating the ratio of the nanomaterials in the hybrid nanofluids, can remarkably influence the thermophysical properties. Different mixture ratios, depending on the properties of each nanomaterial, can affect the TC of hybrid nanofluids. For instance, Osho et al. [43] considered mixture ratios in addition to VF and temperature in measuring the TC of a hybrid nanofluid. The mixture ratios of the nanostructure composed of both Al_2_O_3_ and ZnO were 1:2, 1:1 and 2:1. The maximum improvement in the TC was observed for a mixture ratio of 2:1 (Al_2_O_3_:ZnO), which was equal to 40% for a VF of 1.67%. 

**Figure 4:**
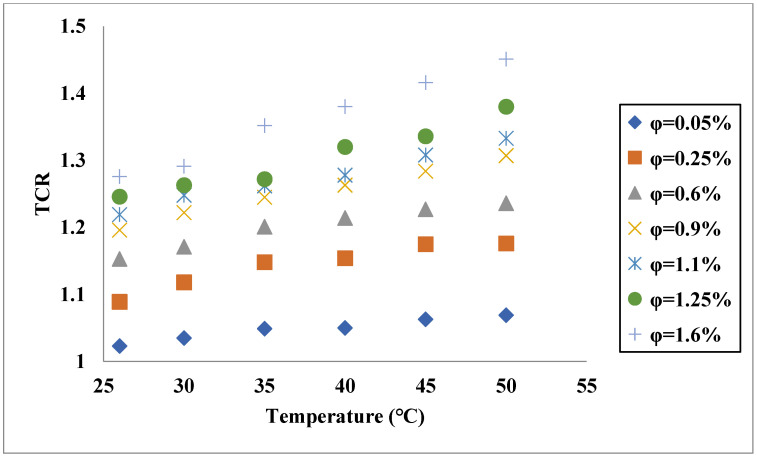
Influences of temperature and VF on TCR of SWCNT-ZnO/EG-water. Adapted from Ref. [49].

**Figure 5:**
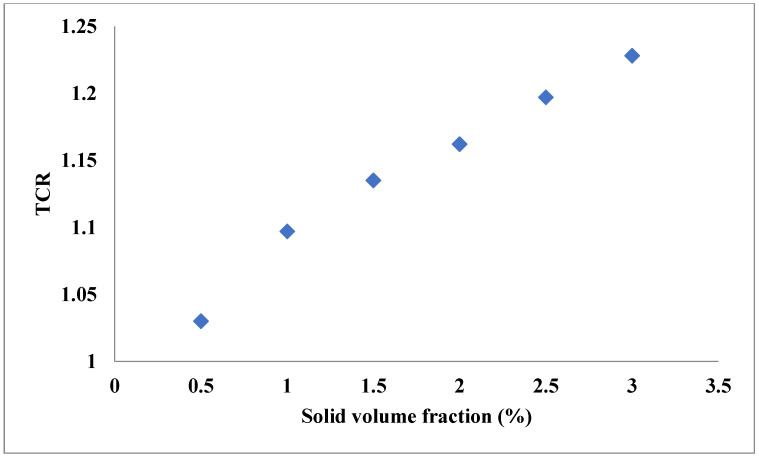
TCR of SiO_2_-TiO_2_/water-EG for different temperatures and VFs and T = 80 °C. Adapted from Ref. [50].

Aside from the single base fluid, a solution of two or more fluids could be applied as the base fluid of hybrid nanofluids [44,45,46,47]. The dispersion of hybrid nanostructures in these types of fluids leads to TC enhancement [48]. Esfe et al. [49] assessed the influences of both concentration and temperature on the TC of a hybrid nanofluid composed of Single-Walled Carbon Nanotube (SWCNT)-ZnO and EG-water as the base fluid. Similar to the hybrid nanofluids with a single base fluid, they found that TCR increased by increases of the temperature and VF of solid structures, as shown in Figure 4. In addition, according to the sensitivity analysis, it was deduced that the sensitivity of TC enhancement was more remarkable to VF compared with the temperature. In another work [50], the TC of a nanofluid composed of SiO_2_-TiO_2_ nanostructures in water-EG was investigated. It was noticed that TCR had higher values in cases of increasing both temperature and solid fraction, and its value could exceed 1.2 at the highest VF and temperature, as shown in Figure 5. In another work [51], the TC of antifreeze-based nanofluid (Go-CuO/EG-water) was measured under different conditions by varying the temperature and VF of solids. Similar to the previously mentioned works, they found that TCR increased by increasing the VF and temperature. In the maximum considered temperature and VF, 50 °C and 1.6%, respectively, the TCR exceeded 1.4. In another research carried out by Esfe et al. [52], the TC of MWCNT-MgO/water-EG nanofluid was investigated in the temperature and concentration ranges of 30–50 °C and 0.015–0.96%, respectively. Despite the low concentrations of the nanostructures, around a 22% improvement in the TC was observed at the maximum temperature and concentration. This noticeable enhancement in the TC by adding the hybrid nanostructures is mainly due to the higher TC of the solid phase compared with the binary base fluid.

Hybrid nanofluids are not restricted to the ones containing two dissimilar materials. Cakmak et al. [53] prepared a hybrid nanofluid composed of three materials as nanostructures, including reduced graphene oxide (rGO), Fe_3_O_4_ and TiO_2_ and EG as the base fluid. The ranges of concentrations and temperatures considered in their study were 0.01–0.25% mass and 25–60 °C, respectively. An improvement of 13.3% in the TC at temperature and concentration of 60 °C and 0.25% wt was observed compared with EG. In another work [54], the TC of a ternary hybrid nanofluid, MWCNT-TiO_2_-ZnO/water-EG, was measured in temperature and VF ranges of 25–50 °C and 0.1–0.4%, respectively. At the maximum temperature and VF, the TC enhancement was around 10%. In Table 1, the works on the hybrid nanofluids’ TC are summarized.

### Proposed Models for Thermal Conductivity

Different techniques are employable to estimate the characteristics of hybrid nanofluids. In several studies, mathematical correlations have been used to estimate hybrid nanofluids’ TC with relatively high precision and reliability. Taherialekouhi et al. [29] proposed a correlation based on the VF of hybrid structures and temperature to model the TCR of a hybrid nanofluid composed of GO-Al_2_O_3_ and water. The ranges of concentration and temperature were 0.1–1% and 25–50 °C, respectively. The highest deviation of the model was 1.598%, showing remarkable accuracy. In another work, the TCR of CNT-SiO_2_/water nanofluid in different temperatures, concentrations and mass fractions of solid materials were modeled by considering all of these variables. The maximum deviation of their model, based on the correlation, was 8.3%. In another research, Esfe et al. [34] applied a correlation for the TCR of EG-based nanofluid containing SWCNT-MgO nanostructures with an R-squared value of around 0.993. 

Despite the advantage of correlation in terms of simplicity to use, the exactness of these models is relatively lower compared with the models based on intelligent techniques, including artificial neural networks (ANNs). By using artificial intelligent methods, models with improved exactness can be developed due to the more complex structure of these networks. These models are generally developed on the basis of simulating the processes of human intelligence by machines. Models based on artificial intelligence are applicable for different purposes related to nanofluids. By these methods, it is possible to model the heat transfer of nanofluidic flows, the performance of the systems utilizing nanofluids and the properties of the fluids containing nanomaterials. In modeling with intelligent methods, it is crucial to consider some factors, including the architecture of the model, input and functions, to reach the outputs with the highest possible accuracy. 

Esfe et al. [35] evaluated the performance of a correlation and ANN in modeling the TCR of SiO_2_-MWCNT/EG and observed that the R-squared of the indicated models were equal to 0.9864 and 0.9981, respectively. In another work [49], correlation and ANN were compared in modeling the TCR of SWCNT-ZnO/EG-water. The obtained values of R-squared for the mentioned models were 0.9918 and 0.9972, respectively. Efficiency of the ANNs in modeling is dependent on different elements, such as applied function and the network architecture. In this regard, to propose an ANN-based model with the highest accuracy in predicting the TC of hybrid nanofluids, testing different architectures and functions would be useful. In a work performed by Vafaei et al. [57], 6, 8, 10 and 12 neurons were tested in the hidden layer (HL) of the applied network proposed for TC modeling of MgO-MWCNT/EG nanofluid. They found that using 12 neurons led to a minimum error, and the predicted data were nearer to the corresponding experimental quantities in comparison with the networks with other numbers of neurons. Aside from the number of neurons, applied functions and the number of HL influence the exactness and performance of ANN-based models. Safaei et al. [58] tested different architectures to find the best network for modeling the TC of ZnO-TiO_2_/EG nanofluid. They found that the most accurate model was obtained using 2 HLs with six and five neurons in the first and second layers, respectively, where the transfer functions in these layers were tan-sig and log-sig, respectively. In addition to the architecture, applied optimization methods in the ANN-based models affect the exactness of the models. Alarifi et al. [59] applied two optimization approaches, GA and PSO, in the models based on ANFIS used for TC of Al_2_O_3_-MWCNT/oil. They found that employing the ANFIS-PSO model led to a lower value of the mean square error compared with ANFIS-GA, which means that by using the PSO algorithm as the optimization approach, the hyperparameters of the models, which significantly affect the error of the predicted values, could be obtained more properly. 

Similar to the hybrid nanofluids with a single base fluid, several models have been proposed for the hybrid ones with base fluids composed of two or more fluids. As an example, Nabil et al. [50] proposed a correlation to model TCR of SiO_2_-TiO_2_/EG-water. The highest value of deviation between the obtained data by correlation and experimental measurement was around 4.6%, showing acceptable exactness of the correlation. In another work, Kakavandi et al. [44] proposed a correlation for the TC modeling of MWCNT-SiC/water EG with a maximum deviation of 1.58%. Rostami et al. [51] applied both correlation and ANN to model the TCR of a hybrid nanofluid composed of binary base fluid (Go-CuO/EG-water). They found that applying ANN led to closer prediction to the experimental values, as was expected, due to its more complex structure and estimation procedures compared with the correlation. In a work done by Akhgar et al. [24], correlation and ANN with different numbers of neurons in HL in range the of 6–31 were tested for modeling the TC of MWCNT-TiO_2_/water-EG nanofluid by considering temperature and VF in the inputs. They found that utilizing eight neurons in the HL led to the network with the highest accuracy, with the maximum error of 2.1%, while the highest error in the case of applying the correlation was 2.72%. In another research [48], the TC of Cu-TiO_2_/water-EG nanofluid was modeled by applying different architectures of ANN. One and two HLs were tested with the 1 to 10 neurons in each layer. They observed that using two layers with five neurons in each one resulted in the highest exactness, with an MSE of 2.62 × 10^−5^. Since the utilization of more hidden layers enables the network to model the complex systems with higher accuracy, this finding was expected; however, in the case of using more hidden layers, the possibility of overfitting is increased. 

There are some models that are applicable for more than one nanofluid. In these cases, it is necessary to consider more inputs compared with the models used for a single case of hybrid nanofluids. In a study by Pourrajab et al. [60], different methods were used to propose models for the TC of hybrid nanofluids with water, EG and the mixture of water and EG as the base fluids. In their work, Locally Weighted Linear Regression (LWLR), Linear Genetic Programming (LGP), Gene Expression Programming (GEP) and some empirical correlations were used for comparison. Aside from temperature and VF, which are commonly used in other studies, the density of materials, their mean size and TC and mixture ratio of water and EG were used as inputs. Root Mean Squared Error (RMSE) of training datasets for the proposed models by LWLR, LGP and GEP were 0.011, 0.0279 and 0.044, respectively. These RMSE values demonstrated higher exactness of the model based on LWLR compared with the others. Considering the effect of the mixture ratio on the TC of hybrid nanofluids, it must be used as one of the inputs in the cases where a model is developed for various mixture ratios. For instance, in [43] different approaches were used to propose a model for TC of Al_2_O_3_-ZnO/water nanofluid in different temperatures, VFs and mixture ratios of the nanostructures. They used ANN, ANFIS and correlation for this purpose and found that applying ANFIS resulted in the highest accuracy. In a work performed by Jamei et al. [61], three methods, including genetic programming (GP), multilinear regression (MLR) and model tree (MT), were used to predict the TC of EG-based hybrid nanofluids. In their models, besides temperature and VF, the sizes of particles in addition to their densities were utilized as inputs. Among the used methods, GP led to the best performance that was followed by MT and MLR, respectively. Table 2 demonstrates the models provided for the TC of hybrid nanofluids.

## 4. Specific Heat

**Figure 6:**
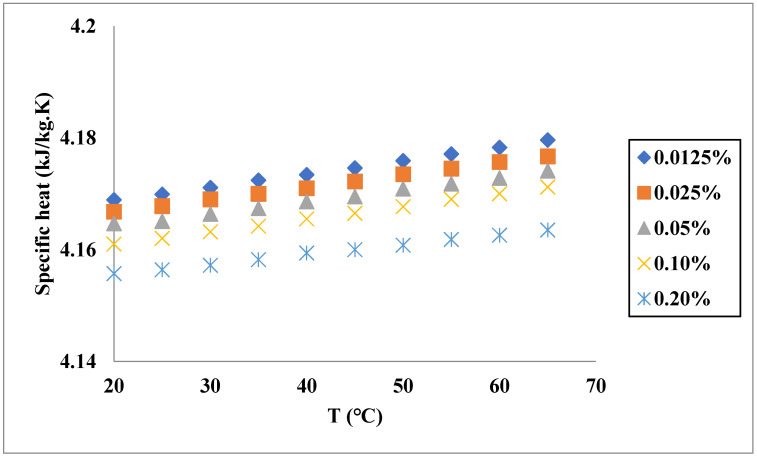
SH variation with temperature of Cu-Al_2_O_3_/water for different solid fractions. Adapted from Ref. [62].

**Figure 7:**
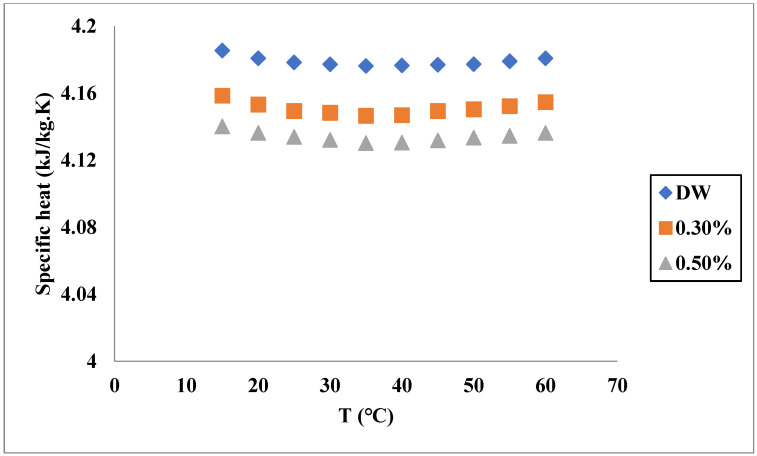
SH variation with temperature of MgO-TiO_2_/distilled water for different solid fractions. Adapted from Ref. [63].

Another thermophysical property of the fluids affecting their heat transfer is specific heat capacity. Similar to the TC, specific heat (SH) is influenced by several factors. In a study conducted by Çolak et al. [62], the SH of Cu-Al_2_O_3_/water nanofluid was investigated at different temperatures and VFs of solids in ranges of 20–65 °C and 0.01255–0.2%, respectively. As illustrated in Figure 6, the SH gradually increased by increasing the temperature, while the increase in the solid VF reduced the SH. In another work [63], the effects of these parameters on the SH were investigated for a MgO-TiO_2_/distilled water hybrid nanofluid. As shown in Figure 7, the increase in temperature reduces the SH to their minimum values, while a further increase in temperature causes an increase in the SH. Similar to the previously mentioned nanofluid, the increase in solid VF results in lower SH in all temperatures. The trend of variations in the SH of the nanofluids in different concentrations was similar to the base fluid since it is the main component of the nanofluid. Gao et al. [64] experimentally investigated the SH of GO-Al_2_O_3_/water nanofluid and observed that a higher solid fraction caused more reduction in the SH. The highest reduction ratio in the SH was observed at the lowest temperature (20 °C) and the highest mass fraction of solids (0.15% wt), which was equal to 7%. More reduction at a higher concentration can be attributed to the lower SH of solid materials in comparison with the liquids used as the base fluid. 

Aside from temperature and solid fraction, other factors, such as the size of particles and their types, influence the reduction in SH. For instance, Tiwari et al. [65] measured the SH of different hybrid nanofluids, including CuO-MWCNT/water, MgO-MWCNT/water and SnO_2_-MWCNT/water, in different solid fractions, temperature and sizes of metal oxide particles (20 to 50 nm). They observed that in cases of the lower size of particles, a higher decrease in the SH occurred, which was attributed to the reduction in the density of the nanofluids due to the increase in particles size. The highest reduction in the SH of the nanofluids was 15.09% at a temperature of 25 °C and a mean size of 20 nm. 

In addition to the nanofluids with two dissimilar nanomaterials, the SH of ternary hybrid nanofluids with three nanomaterials has been investigated. In a study conducted by Mousavi et al. [66], the SH of a ternary hybrid nanofluid with various mass ratios of the particles CuO-MgO-TiO_2_ was measured. The mass ratios of the particles were (A: 33.4%:33.3%:33.3%, B: 50%:25%:25%, C: 60%:30%:10%, D: 25%:50%:25% and E: 25%:25%:50%). The considered VF and temperature were 0.1–0.5% and 15–60 °C, respectively. The measured values of the SH revealed that it had a reducing trend up to 35 °C, afterward it had an increasing trend by increasing the temperature. The maximum decrease in the SH of the nanofluids was noticed for the C type. Consequently, it was concluded that the material specifications of the nanostructures influenced the SH of the hybrid nanofluid. The influence of the materials on the SH of the hybrid nanofluids has been observed in other studies [67]. Table 3 summarizes the works on the SH of hybrid nanofluids.

### Proposed Models for Specific Heat

Compared with the TC and dynamic viscosity, there are few works on the modeling of SH. This property of the hybrid nanofluids has been modeled and predicted by different techniques, such as correlation, support vector machines and ANN [68,69,70]. For instance, Mousavi et al. [63] proposed a correlation for SH of MgO-TiO_2_/water and found the model reliable since its R-squared was 0.993. Çolak et al. [62] used both correlation and ANN to model the SH of Cu-Al_2_O_3_/water by using temperature and solid fraction. They found that using ANN provided a model with a much lower relative error. Similar to TC, lower errors of the model based on ANN for predicting the SH are mainly due to its more complex structure and determination procedures. 

There are some models with improved comprehensiveness that are applicable for more than one nanofluid. For instance, Tiwari et al. [65] proposed a correlation by considering the density, size and SH of nanoparticles besides temperature and VF for SH modeling of three different nanofluids, including CuO-MWCNT/water, MgO-MWCNT/water and SnO_2_-MWCNT/water. The maximum error of their model was 2.93%, while the average absolute relative error was 0.903%, demonstrating the high exactness of the model. In another work [67], a correlation was proposed to model the SH of water-based hybrid nanofluids containing Al_2_O_3_-TiO_2_ and Al_2_O_3_-Si_2_O_3_ for different temperatures and VFs. In their correlation, densities of water and nanostructure in addition to size and concentration were used for regression; however, the model was not very accurate, and the average deviation was 11%, and they concluded that to reach an accurate prediction, it would be preferred not to use the unified model. In Table 4, the results of the works on SH estimation of hybrid nanofluids are provided. 

## 5. Dynamic Viscosity

Dynamic viscosity (DV) is an important property of heat transfer fluid that affects the fluid heat transfer characteristics. Generally, nanoparticles suspension in the liquid increases the fluid DV, which is a function of different factors [71,72,73,74,75]. In a study done by Motahari et al. [76], the impacts of the solid fraction and temperature on the DV of MWCNT-SiO_2_/20W50 oil were assessed. The considered solid fraction and temperature ranges in their work were 0.05–1% and 40–100 °C, respectively. They found that at the highest temperature and solid fraction, DV increased up to 171% compared with the base fluid. In another work [77], the DV of graphene-NiO/coconut oil was investigated in different solid fractions and temperatures. Similar to the previous study [76], it was observed that DV increased by increasing the solid fraction while it decreased by increasing the temperature. The enhancement in the DV of the nanofluid at a temperature of 120 °C and solid weight fraction of 0.5% was 28.49%. In addition to the abovementioned factors, the material of the nanostructures can affect the DV of the hybrid nanofluids. As an example, Ghaffarkhah et al. [78] investigated the DV of oil-based hybrid nanofluids with various materials, including MWCNT-SiO_2_, MWCNT-Al_2_O_3_ and MWCNT-TiO_2_. It was found that the impact of the material was very low, and the maximum improvements in the DV of the hybrid nanofluids with the mentioned materials were 13.015%, 13.618% and 12.559%, respectively. However, in a work by Dalkılıç et al. [79] on the DV of SiO_2_-graphite/water, it was found that SiO_2_ particles had more effect on the DV compared with graphite. The maximum increase in the DV was 36.12%, which was obtained for a solid fraction of 2%. 

The surfactant of the hybrid nanofluids and their concentration influence the DV. Ma et al. [80] investigated the impacts of surfactant on the DV of Al_2_O_3_-TiO_2_/water and Al_2_O_3_-CuO/water nanofluids and found that the increase in the PVP, used as a surfactant, led to the enhancement in the DV of the nanofluid. In addition, it was noticed that an increase in the concentration of the surfactant to more than 0.02% wt caused a significant increase in the DV. 

**Figure 8:**
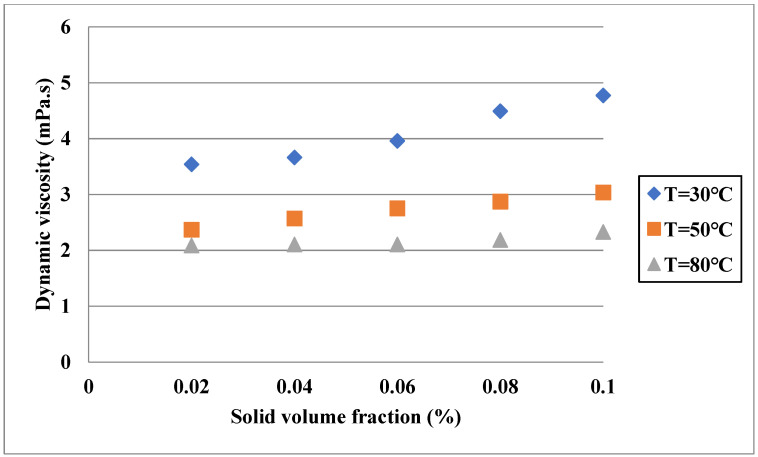
Effect of volume concentration on DV of TiO_2_-Al_2_O_3_/water-EG. Adapted from Ref. [81].

The DV of hybrid nanofluids with the base fluids composed of two liquids has been considered in some works. For instance, Urmi et al. [81] carried out a study on the DV of TiO_2_-Al_2_O_3_/water-EG nanofluids considering the impacts of solid fraction and temperature. As shown in Figure 8, similar to single base fluid hybrid nanofluids, the DV increased by increasing the VF and decreased by increasing the temperature. The highest value of relative DV was observed at a temperature of 80 °C and a VF of 0.1%, which was equal to 161.8%. In another work [82], the DV of Al_2_O_3_-CuO/EG-water and Al_2_O_3_-CuO/propylene glycol (PG) was measured in the temperature range of 50–70 °C and solid VF of 0–1.5%. Moreover, the mixture ratio of the base fluid was varied to investigate its effect. It was observed that the increase in the fraction of nanostructures, EG and PG, led to higher DV of the hybrid nanofluids; however, at a temperature of 70 °C, the increase in the fraction of EG from 50% to 55% led to lower dynamic viscosity. 

Aside from the hybrid nanofluids with two dissimilar materials, the DV of the ones with three dissimilar materials has been investigated by some researchers. For instance, Sahoo [83] performed a study on the DV of a ternary hybrid nanofluid. This nanofluid was composed of water as the base fluid and Al_2_O_3_, TiO_2_and SiC nanoparticles. Similar to the conventional hybrid nanofluids, it was observed that the increase in the temperature caused a reduction in the DV while an increase in solid fraction led to higher DV values. In another work [84], the DV of Al_2_O_3_-CuO-TiO_2_/water was measured for temperature and solid fraction ranges of 35–50 °C and 0.01–0.1%, respectively. It was noticed that by increasing the temperature from 35 to 50 °C, the DV decreased by up to 23.64%. In addition, a comparison between the ternary hybrid nanofluid and Al_2_O_3_-CuO/water and Al_2_O_3_-TiO_2_/water revealed a higher DV of the ternary nanofluid. 

**Figure 9:**
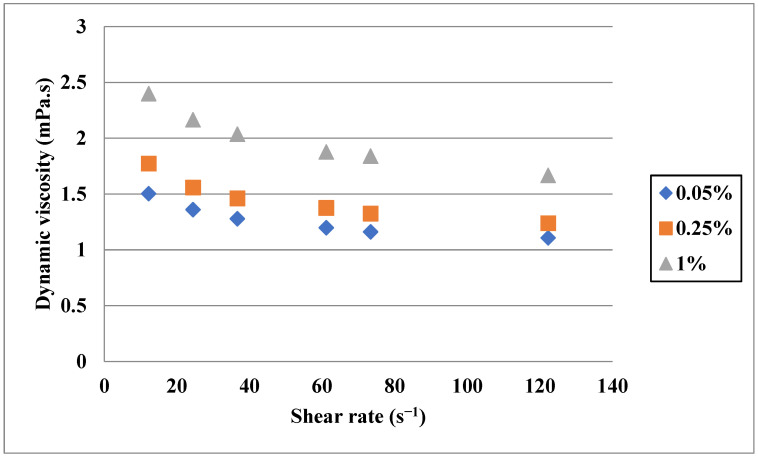
Effect of shear rate on the DV of graphene-SiO_2_/water at a temperature of 25 °C. Adapted from Ref. [90].

Due to the non-Newtonian behavior of some of the nanofluids [85,86], it is crucial to consider the shear rate in measuring and reporting the DV. Contrary to the Newtonian fluids, shear rate affects the DV of non-Newtonian fluids. In Newtonian fluids, the DV is influenced by the stress and the behavior of the fluid under the force can change to more solid or more liquid. In order to find the impacts of stress on the DV of these types of fluids, different shear rates can be applied in the measurement procedure. Esfe [87] measured the DV of MgO-MWCNT/5W50 oil in various solid fractions (0.05–1%), temperatures (5–55 °C and shear rates (665.5–11997 $s^{-1}$). According to their observations, at low temperatures, the DV had relatively high dependency on the shear rate and its value decreased by shear rate; however, at higher temperatures, this dependency diminished. In addition, it was observed that the increase in the VF caused an increase in the DV, while the effect of temperature was the reverse. In another work, the effects of temperature, shear rate and solid fraction on the DV of SiO_2_-MWCNT/10W40 oil were investigated by Nadooshan et al. [88]. It was observed that the nanofluid had non-Newtonian behavior at all temperatures, but the base fluid had non-Newtonian behavior just at high temperatures. Alirezaie et al. [89] investigated the DV of MWCNT (COOH-Functionalized)-MgO/engine oil in a temperature range of 25–50 °C and a shear rate of 670–8700 $s^{-1}$. They found that the nanofluid showed relatively non-Newtonian behavior; however, at high temperatures, it became Newtonian. Similar to previous nanofluids, the DV increases with the increase in the solid fraction. Kazemi et al. [90] investigated the DV of graphene-SiO_2_/water at different shear rates and found that the nanofluid was non-Newtonian. As shown in Figure 9, the DV of the nanofluid increased by increasing the solid fraction and reducing the shear rate. Hybrid nanofluids with binary base fluids may show non-Newtonian behavior. As an example, Bahrami et al. [91] investigated the DV of Fe-CuO/water-EG in different mixture ratios of the base fluid, temperatures, shear rates and solid fractions. They found that the nanofluid showed Newtonian behavior at low solid fractions while it became non-Newtonian at high concentrations. 

In Table 5, the main findings of the works on the DV of hybrid nanofluids are provided. 

### Proposed Models for Dynamic Viscosity

Similar to TC and SH, there are several models for the DV of hybrid nanofluids by using correlations, ANNs, etc. For instance, in a study by Motahari et al. [76] regarding the temperature of solid volume, a correlation was provided for the DV ratio of MWCNT-SiO_2_/20W50 oil with an average deviation of 1.75%. Asadi et al. [72] proposed a simple model for DV considering temperature and solid fraction of MWCNT-MgO-SAE50. In the suggested correlation, the maximum error was around 8%. Urmin et al. [81] proposed a correlation in terms of solid fraction and temperature for the DV of TiO_2_-Al_2_O_3_/water-EG and found that the maximum deviation of the model was 12.7%. In another study [72], a correlation was suggested for the DV ratio of ZnO-Ag/water nanofluid regarding the VF of solid particles. In the proposed correlation, a polynomial of the third degree was used, and the deviation margin of the obtained model was 1.8%. Models based on correlations are employable for ternary hybrid nanofluids. As an example, Sahoo et al. [84] used a correlation in terms of solid fraction and temperature to suggest a model for the DV of Al_2_O_3_-CuO-TiO_2_/water. The maximum deviation of their correlation for estimating the DV of the nanofluid was 1.5%. The models based on the correlation can be improved in terms of comprehensiveness by using more variables as inputs. Since the mixture ratio of the nanomaterials can affect the DV of hybrid nanofluids, similar to the TC, considering it as one of the variables would lead to improvement in the comprehensiveness of the models. For instance, Dalkılıç et al. [79] used the ratio of graphite weight to silica weight in addition to the temperature and solid fraction to provide a model for the DV of SiO_2_-graphite/water for different mixture ratios of the solid materials. The average deviation of the model by using the mentioned variables as input was 6.75%.

Since the nanofluids may have non-Newtonian behavior, other factors in addition to solid fraction and temperature must be used in the models to have more accurate outputs. For instance, Esfe [87] proposed a correlation for the DV of MgO-MWCNT/5W50 oil nanofluid by considering temperature, solid fraction and shear rate. The proposed model had adequate exactness with a maximum error of 8%. Toghraie et al. [86] suggested two models based on correlation and ANN for the DV of a non-Newtonian hybrid nanofluid composed of WO_3_ and MWCNT. In their work, various numbers of neurons were tested to find the optimum structure, and it was found that using 39 neurons in the HL led to the best performance. In addition, they found that ANN leads to higher exactness in comparison with the correlation. In another work [89], the DV of MWCNT (COOH-Functionalized)-MgO/engine oil was modeled by considering the shear rate as one of the inputs and employing correlation and ANN. They found that the R-squared of the models by using the ANN was 0.9973 while it was 0.98 in the case of applying correlation. 

In addition to correlations, other approaches would be useful for proposing more precise models [77]. For instance, Jamei et al. [98] applied advanced Genetic Programming (GP), which was called Multigene Genetic Programming (MGGP), in addition to Multi-variate Linear Regression (MLR) and Gene Expression Programming (GEP) to predict the relative DV of various hybrid nanofluids with Newtonian behavior. The inputs in their models were density and size of the particles, temperature, the DV of the base fluid and the VF of solids. It was noticed that MGGP had the highest exactness with a root mean squared error (RMSE) of 0.05, followed by GEP and MLR with the values of 0.083 and 0.153, respectively. Furthermore, according to the sensitivity analysis, they concluded that the solid fraction, temperature and size of particles were the most influential elements in the relative DV of the investigated hybrid nanofluids. In another work conducted by Ghaffarkhah et al. [78], various approaches, including the Group Method of Data Handling (GMDH), Support Vector Machine (SVM), Radial Basis Function (RBF) and Multi-layer Perceptron (MLP), coupled with different optimization algorithms were used to model the DV of hybrid nanofluids. Comparing the exactness of the proposed models demonstrates that GMDH outperforms other approaches. Table 6 summarizes the works on the estimation of hybrid nanofluids’ DV. 

## 6. Conclusions

The main challenge in improving the thermal performance of a fluid is to improve the working fluid thermophysical properties. In the present work, the thermophysical properties of the hybrid nanofluids, including thermal conductivity, specific heat and dynamic viscosity, as well as their proposed models, are reviewed. The main findings of the study, regarding the questions provided in the methodology section, can be summarized as follows:

- Temperature and solid fraction significantly influence the characteristics of hybrid nanofluids.
- In addition to the abovementioned factors, nanostructure material, mixture ratio and base fluid affect the features of hybrid nanofluids.
- Dispersion of the hybrid nanostructures can notably improve the TC of hybrid nanofluids, which is more remarkable at higher temperatures.
- The DV of hybrid nanofluids increases with an increase in the solid fraction.
- Some of the hybrid nanofluids show Newtonian behavior, while some others are non-Newtonian. At higher solid fractions, there is more tendency toward non-Newtonian behavior.
- Both simple correlations and intelligent methods are applicable for modeling the properties of hybrid nanofluids.
- The exactness of the models proposed for thermophysical properties of a nanofluid is mainly under the influences of method and input variables.
- Intelligent methods, such as ANNs, are preferred in terms of accuracy compared with the correlation for modeling the properties of hybrid nanofluids.
- The accuracy of the intelligent methods can be modified by applying more proper functions and optimization algorithms.

## 7. Future Recommendations

In this review paper, important thermophysical features of the hybrid nanofluids, including the TC, specific heat and DV, and the proposed models were reviewed. Despite the numerous studies in the relevant fields, some suggestions are provided for upcoming studies to reach more desirable outcomes. First of all, the effects of other factors, such as the shape of nanostructures and type of surfactants, can be considered in future work regarding their influence on the thermal performance of the nanofluidic devices [100]. Furthermore, it would be attractive to propose models with higher comprehensiveness by applying more variables as inputs. The features of the nanostructure are among the variables that can develop the models’ applicability. In addition, despite the key role of the mixture ratio of nanomaterials on the thermophysical properties of the hybrid nanofluids, only a few studies have considered this factor for the TC and DV. Future works should consider the mixture ratio with wider ranges for different hybrid nanofluids. In addition, the effect of mixture ratio on the SH of hybrid nanofluids should be considered in future works. 

Furthermore, different data-driven methods, such as SVM, can be used more widely. In addition, novel and efficient optimization algorithms should be used and coupled with data driven approaches to reach the minim deviation in modeling the properties of the hybrid nanofluids. 

## Figures and Tables

**Table 1:** Findings of the works on the TC of hybrid nanofluids.

Reference	Nanostructures	Base Fluid	Important Findings
Akhgar et al. [46]	TiO_2_-MWCNT	Water-EG	Up to 38.7% enhancement in TC at temperature and VF of 50 °C and 1%, respectively.
Aparna Z. et al. [25]	Al_2_O_3_-Ag	Water	Compared with water, up to 23.82% enhancement in the TC of nanofluid was observed at temperature of 325 K.
Esfe et al. [27]	MWCNT-SiO_2_	EG	Up to 20.1% enhancement in the TC of nanofluid compared with EG.
Esfahani et al. [28]	ZnO-Ag	Water	TCR of the nanofluid was higher than 1.25 at temperature of 50 °C and VF of 2%.
Toghraie et al. [32]	ZnO-TiO_2_	EG	Up to 32% improvement in TC at temperature of 50 °C and VF of 3.5%.
Esfe et al. [31]	SWCNT-Al_2_O_3_	EG	Up to 41.2% enhancement in TC at temperature of 50 °C and VF of 2.5%.
Zadkhast et al. [33]	MWCNT-CuO	Water	Up to 30.38% enhancement in TC at temperature and VF of 50 °C and 0.6%, respectively.
Esfe et al. [34]	SWCNT-MgO	EG	More than 35% enhancement in TC at temperature and VF of 50 °C and 0.55%, respectively.
Bakhtiari et al. [26]	TiO_2_-Gr	Water	Up to 27.84% increase in TC was observed in 75 °C and VF of 0.5%.
Taherialekouhi et al. [29]	GO-Al_2_O_3_	Water	TC of nanofluid increases by up to 33.9% in case of existence of the nanostructures.
Esfe et al. [35]	SiO_2_-MWCNT	EG	Increase in temperature and VF of solid phase brings out higher thermal conductivity ratio.
Esfe et al. [38]	SiO_2_-DWCNT	EG	Up to 38% improvement in TC compared with EG.
Singh et al. [30]	Go-CuO	Distilled water	30% enhancement in TC at 60 °C and 0.3% wt concentration.
Soltani et al. [36]	WO_3_-MWCNT	Engine oil	Maximum increase in TC was 19.85% compared with the base fluid.
Pourrajab et al. [39]	Ag-MWCNT	Water	By dispersing the nanostructures, up to 47.3% improvement in TC was reachable.
Dalkılıç et al. [41]	CNT-SiO_2_	Water	Mixture ratio of solid components affects the TC enhancement of the nanofluid.
Moghadam et al. [55]	GO-TiO_2_	Water	Maximum enhancement of TC was 32.8%.
Tiwari et al. [42]	CeO_2_-MWCNT	Water	Surfactant and sonication time affect TCR of the hybrid nanofluid.
Trinh et al. [40]	Gr-CNT	EG	Up to 50% TC improvement compared with the base fluid in 0.07% VF.
Esfe et al. [48]	Cu-TiO_2_	Water-EG	TCR exceeds 1.4 at 60 °C and VF of 2%.
Kakavandi et al. [44]	MWCNT-SiC	Water-EG	Up to 33% increase in the TC of nanofluid at temperature equal to 50 °C and VF of 0.75%.
Wole-Osho et al. [43]	Al_2_O_3_-ZnO	Water	Mixture ratio of 2:1 (Al_2_O_3_: ZnO) leads to the highest TC compared with 1:2 and 1:1.
Esfe et al. [49]	SWCNT-ZnO	EG-water	Sensitivity of TC to the VF was more notable compared with temperature.
Esfe et al. [52]	MWCNT-MgO	Water-EG	Around 22% enhancement in TC was observed at temperature and VF of 50 °C and 0.96%.
Leong et al. [47]	Cu-TiO_2_	EG-water	Up to 9.8% enhancement in TC in mass fraction of 0.8%.
Rostamian et al. [45]	CuO-SWCNT	EG-water	Around 35% enhancement in TC at temperature equal to 50 °C and VF of 0.75%.
Esfe et al. [37]	graphene-SiO_2_	Water	Around 36% enhancement in the TC was achievable in temperature of 50 °C and VF of 1%.
Nabil et al. [50]	SiO_2_-TiO_2_	Water-EG	TCR can exceed 1.2 at the maximum considered temperature and VF.
Rostami et al. [51]	Go-CuO	EG-water	At the highest temperature and solid VF, TCR exceeds 1.4.
Moradi et al. [56]	TiO_2_-MWCNT	EG-water	Around 34% enhancement in TC at temperature of 60 °C and VF of 1%.
Cakmak et al. [53]	rGO-Fe_3_O_4_-TiO_2_	EG	Around 13% enhancement in TC at temperature of 60 °C and mass concentration of 0.25%.
Boroomandpour et al. [54]	MWCNT-TiO_2_-ZnO	Water-EG	Around 10% enhancement in TC at the maximum considered temperature and VF.

**Table 2:** Proposed models for TC of hybrid nanofluids.

Reference	Nanostructures	Base Fluid	Method	Important Findings
Aparna Z. et al. [25]	Al_2_O_3_-Ag	Water	Correlation	R-squared of the proposed correlation was 0.9748.
Bakhtiari et al. [26]	TiO_2_-Gr	Water	Correlation	Margin error of the model was 1.44%.
Moghadam et al. [55]	GO-TiO_2_	Water	Correlation	The correlation was able to model TC with acceptable exactness.
Taherialekouhi et al. [29]	GO-Al_2_O_3_	Water	Correlation	The model highest deviation was 1.598%.
Esfe et al. [34]	SWCNT-MgO	EG	Correlation	R-squared of the proposed correlation was around 0.993.
Toghraie et al. [32]	ZnO-TiO_2_	EG	Correlation	The model highest deviation was 1.74%.
Moradi et al. [56]	TiO_2_-MWCNT	EG-water	Correlation	The model highest deviation was 2.72%.
Zadkhast et al. [33]	MWCNT-CuO	Water	Correlation	TCR of the nanofluid was predicted with acceptable accuracy by using the proposed correlation.
Esfahani et al. [28]	ZnO-Ag	Water	Correlation	Deviation margin of the model was 1.3%.
Esfe et al. [31]	SWCNT-Al_2_O_3_	EG	Correlation and ANN	Maximum deviations of the models by applying correlation and ANN were 2.6% and 1.94%, respectively.
Safaei et al. [58]	ZnO-TiO_2_	EG	Correlation and ANN	ANN-based model with optimal geometry had much higher accuracy compared with the correlation.
Esfe et al. [27]	MWCNT-SiO_2_	EG	Correlation and ANN	Using two HLs with four neurons in each layer led to the highest exactness of the ANN-based model with R-squared of 0.9989.
Dalkılıç et al. [41]	CNT-SiO_2_	Water	Correlation	The model highest deviation was 8.3%.
Esfe et al. [35]	SiO_2_-MWCNT	EG	Correlation and ANN	R-squared of the models by using correlation and ANN were 0.9864 and 0.9981, respectively.
Esfe et al. [38]	SiO_2_-DWCNT	EG	Correlation and ANN	The number of HL, applied functions and number of neurons affect the exactness of the ANN-based model.
Esfe et al. [48]	Cu-TiO_2_	Water-EG	Correlation and ANN	MSE values of the correlation and optimum ANN were 1.33 × 10^−4^ and 2.62 × 10^−5^, respectively.
Esfe et al. [49]	SWCNT-ZnO	EG-water	Correlation and ANN	R-squared of the models on the basis of correlation and ANN were 0.9918 and 0.9972, respectively.
Esfe et al. [37]	graphene-SiO_2_	Water	Correlation	R-squared of the model was 0.99.
Pourrajab et al. [39]	Ag-MWCNT	Water	Correlation	R-squared of the model was 0.992.
Kakavandi et al. [44]	MWCNT-SiC	Water-EG	Correlation	The maximum deviation of the model was 1.58%.
Vafaei et al. [57]	MgO-MWCNT	EG	Correlation and ANN	Using 12 neurons in the HL of the network leads to higher accuracy compared with the cases of utilizing 6, 8 and 10 neurons.
Alarifi et al. [59]	Al_2_O_3_-MWCNT	Oil	ANFIS-PSO and ANFIS-GA	Using ANFIS-PSO led to higher accuracy compared with ANFIS-GA.
Nabil et al. [50]	SiO_2_-TiO_2_	Water-EG	Correlation	Maximum deviation was lower than 5%.
Rostamian et al. [45]	CuO-SWCNT	EG-water	Correlation and ANN	Maximum deviation in case of applying ANN was 0.544%.
Akhgar et al. [24]	MWCNT-TiO_2_	Water-EG	Correlation and ANN	Using ANN in optimal architecture led to maximum error of 2.1% while this value was 2.72% when correlation was applied.
Akhgar et al. [46]	TiO_2_-MWCNT	Water-EG	Two correlations	R-squared of the proposed correlations in modeling TC of the nanofluid was around 0.99.
Pourrajab et al. [60]	Different hybrid nanostructures	Water, EG and different mixture of these fluids	LWLR, LGP and GEP	Using LWLR leads to the model with the highest accuracy.
Wole-Osho et al. [43]	Al_2_O_3_-ZnO	Water	Correlation, ANN and ANFIS	Using ANFIS for modeling led to the highest accuracy.
Rostami et al. [51]	Go-CuO	EG-water	Correlation and ANN	Compared with the correlation, applying ANN leads to higher accuracy.
Cakmak et al. [53]	rGO-Fe_3_O_4_-TiO_2_	EG	Correlation	Depending on the temperature, R-squared of the proposed model was in range of 0.954 and 0.99.
Jamei et al. [61]	Different nanostructures	EG	GP, MT and MLR	Using GP led to the highest accuracy followed by MT and MLR.

**Table 3:** Findings of the works on the SH of hybrid nanofluids.

Reference	Nanostructures	Base Fluid	Important Findings
Çolak et al. [62]	Cu-Al_2_O_3_	Water	Reduction in SH of the hybrid nanofluid was noticed by an increase in the solid VF.
Mousavi et al. [63]	MgO-TiO_2_	Water	Increase in temperature reduced the SH to their minimum values while further increase leads to increase in SH.
Gao et al. [64]	GO-Al_2_O_3_	Water	Up to 7% reduction ratio in SH of the nanofluid at temperature of 20 °C and mass fraction of 0.15%.
Tiwari et al. [65]	CuO-MWCNT, MgO-MWCNT and SnO_2_-MWCNT	Water	Higher reduction in the SH was observed in lower sizes of metal oxide particles.
Mousavi et al. [66]	CuO-MgO-TiO_2_	Water	Mass fraction ratio of the nanostructures affects SH of the hybrid nanofluid.
Moldoveanu et al. [67]	Al_2_O_3_-TiO_2_ and Al_2_O_3_-Si_2_O_3_	Water	Dispersion of Al_2_O_3_-TiO_2_in the base fluid led to higher reduction in the SH.

**Table 4:** Proposed models for the SH of hybrid nanofluids.

Reference	Nanostructures	Base Fluid	Method	Important Findings
Çolak et al. [62]	Cu-Al_2_O_3_	Water	Correlation and ANN	ANN provided a model with much lower relative error compared with the correlation.
Mousavi et al. [63]	MgO-TiO_2_	Water	Correlation	R-squared of the correlation was 0.993.
Tiwari et al. [65]	CuO-MWCNT, MgO-MWCNT and SnO_2_-MWCNT	Water	Correlation	The maximum deviation of their model was 2.93%.
Mousavi et al. [66]	CuO-MgO-TiO_2_	Water	Correlation	The deviation of the model was around 1%.
Moldoveanu et al. [67]	Al_2_O_3_-TiO_2_and Al_2_O_3_-Si_2_O_3_	Water	Correlation	The average deviation was 11%.

**Table 5:** Findings of the works on the DV of hybrid nanofluids.

Reference	Nanostructures	Base Fluid	Important Findings
Asadi et al. [72]	MWCNT-MgO	SAE50 oil	Up to 65% increase in DV at temperature of 40 °C and solid fraction of 2%.
Alarifi et al. [73]	TiO_2_-MWCNT	5W50 oil	Up to 42% increase in DV at temperature of 50 °C and solid fraction of 2%.
Asadi et al. [75]	CuO-TiO_2_	Water	Increase in the DV by increase in the solid fraction.
Goodarzi et al. [74]	ZnO-MWCNT	SAE 10W40	Up to around 100% increase in the DV was observed.
Senniangiri et al. [77]	Gr-NiO	Coconut oil	28.49% enhancement in DV at temperature and mass fraction of 120 °C and 0.5%, respectively.
Esfe [87]	MgO-MWCNT	5W50 oil	Dependency of viscosity on shear rate decreased at higher temperatures.
Dalkılıç et al. [79]	SiO_2_-graphite	Water	Up to 36.12% increase in DV in VF of 2%.
Kazemi et al. [90]	graphene-SiO_2_	Water	Increase in the shear rate caused reduction in DV.
Ma et al. [80]	Al_2_O_3_-TiO_2_ and Al_2_O_3_-CuO	Water	Increase in the surfactant concentration led to an increase in the DV.
Motahari et al. [76]	MWCNT-SiO_2_	20W50 oil	Up to 177% increase in the DV was observed at a solid fraction of 1%.
Ruhani et al. [71]	ZnO-Ag	Water	Relative DV of the nanofluid was around 1.75 at a solid fraction of 2% and temperature of 25 °C.
Alirezaie et al. [89]	MWCNT (COOH-Functionalized)-MgO	Engine oil	At low temperatures, the behavior of the nanofluid was non-Newtonian; however, it becomes Newtonian at high temperatures.
Urmi et al. [81]	TiO_2_-Al_2_O_3_	Water-EG	Up to 161.8% enhancement in the relative DV of hybrid nanofluid.
Nadooshan et al. [88]	SiO_2_-MWCNT	10W40	DV increased by an increase in the solid fraction.
Toghraie et al. [86]	WO_3_-MWCNT	Engine oil	Reduction in the DV of nanofluid by increasing the temperature.
Kumar et al. [82]	Al_2_O_3_-CuO	EG-water and PG-water	Increase in DV of hybrid nanofluid with solid fraction increase.
Ghaffarkhah et al. [78]	Different materials	Transformer oil	Effect of the nanostructure material on the maximum enhancement of the DV was very low.
Sahoo [83]	Al_2_O_3_, TiO_2_ and SiC	Water	Increase in DV by temperature reduction and solid fraction increase.
Sahoo et al. [84]	Al_2_O_3_, TiO_2_ and CuO	Water	Up to 23.64% reduction in DV of the nanofluid by increasing temperature from 35 to 50 °C.
Bahrami et al. [91]	Fe-CuO	Water-EG	The nanofluid showed Newtonian behavior at low concentrations while it was non-Newtonian at high solid fractions.
Nabil et al. [50]	TiO_2_-SiO_2_	Water-EG	Up to 80% increase in relative DV at VF of 3%.
Afrand et al. [92]	SiO_2_-MWCNT	SAE40	Maximum enhancement of the DV in VF of 1% was 37.4%.
Asadi et al. [93]	MWCNT-ZnO	Engine oil	Up to 45% increase in DV in solid fraction of 1%.
Huminic et al. [94]	Fe-Si	Water	Increase in DV by increase in the solid fraction of nanofluid.
Esfe et al. [95]	MWCNT-SiO_2_	SAE40	Up to 30.2% enhancement in DV at VF of 1%.
Solatani et al. [96]	MgO-MWCNT	EG	Up to 168% enhancement in DV by increasing the solid fraction from 0.1% to 1%.
Aghaei et al. [97]	CuO-MWCNT	SAE5W50	Up to 35.52% increase in the DV by increasing the solid fraction from 0.05% to 1%.

**Table 6:** Proposed models for the DV of hybrid nanofluids.

Reference	Nanostructures	Base Fluid	Method	Important Findings
Esfe [87]	MgO-MWCNT	5W50 oil	Correlation	The model highest error was 8%.
Motahari et al. [76]	MWCNT-SiO_2_	20W50 oil	Correlation	The model mean deviation was 1.75%.
Alarifi et al. [73]	TiO_2_-MWCNT	5W50 oil	Correlation	The model highest error was 4%.
Ruhani et al. [71]	ZnO-Ag	Water	Correlation	Deviation margin of the model was 1.8%.
Goodarzi et al. [74]	ZnO-MWCNT	SAE 10W40	Correlation	R-squared of the correlation was around 0.997.
Asadi et al. [72]	MWCNT-MgO	SAE50 oil	Correlation	The model highest error was 8%.
Urmin et al. [81]	TiO_2_-Al_2_O_3_	Water-EG	Correlation	Maximum deviation of the model was 12.7%.
Sahoo et al. [84]	Al_2_O_3_, TiO_2_ and CuO	Water	Correlation	Maximum deviation of the model was 1.5%.
Dalkılıç et al. [79]	SiO_2_-graphite	Water	Correlation	Average deviation of the model was 6.75%.
Afrand et al. [92]	SiO_2_-MWCNT	SAE40	Correlation	Maximum deviation of the model was 0.75%.
Toghraie et al. [86]	WO_3_-MWCNT	Engine oil	Correlation and ANN	R-squared of the models based on ANN and correlation were around 0.9998 and 0.9994, respectively.
Ghaffarkhah et al. [78]	Different materials	Transformer oil	GMDH, SVM, MLP and RBF with different optimization methods	GMDH outperforms the other methods in predicting DV of the hybrid nanofluids.
Jamei et al. [98]	Different materials	Different oils	MLR, MGGP and GEP	Applying MGGP led to the highest exactness in prediction of relative DV.
Alirezaie et al. [89]	MWCNT (COOH-Functionalized)-MgO	Engine oil	Correlation and ANN	R-squared of the ANN and correlation were 0.9973 and 0.98, respectively.
Sahoo [83]	Al_2_O_3_, TiO_2_ and SiC	Water	Correlation	R-squared of the model in term of solid fraction was 0.9887.
Nabil et al. [50]	TiO_2_-SiO_2_	Water-EG	Correlation	Maximum deviation of the model was 9.5%.
Esfe et al. [95]	MWCNT-SiO_2_	SAE40	Correlation	Maximum deviation of the model was 1.2%.
Afrand et al. [99]	MWCNT-SiO_2_	AE40	Correlation and ANN	The deviation margins of the correlation and ANN were 4% and 1.5%, respectively.
Aghaei et al. [97]	CuO-MWCNT	SAE5W50	Correlation and ANN	R-squared values were 0.9998 and 0.998 in cases of employing ANN and correlation.

Using hybrid models have been implemented to numerical solution of nanofluid flow problems. Adapted from Ref. [89] and Ref. [90].

## Data Availability

Not applicable.

## Nomenclature

ANFIS	Adaptive Neuro-Fuzzy Inference System
ANN	Artificial Neural Network
CNT	Carbon Nano Tube
DV	Dynamic Viscosity
GA	Genetic Algorithm
GEP	Gene Expression Programming
GMDH	Group Method of Data Handling
GP	Genetic Programming
HL	Hidden Layer
LGP	Linear Genetic Programming
LWLR	Locally Weighted Linear Regression
MLR	Multilinear Regression
MSE	Mean Squared Error
MT	Model Tree
PSO	Particle Swarm Optimization
RBF	Radial Basis Function
RMSE	Root Mean Squared Error
SH	Specific Heat
SVM	Support Vector Machine
TC	Thermal Conductivity
TCR	Thermal Conductivity Ratio
VF	Volume Fraction
